# Associated bacterial microbiome responds opportunistic once algal host *Scenedesmus vacuolatus* is attacked by endoparasite *Amoeboaphelidium protococcarum*

**DOI:** 10.1038/s41598-022-17114-1

**Published:** 2022-08-01

**Authors:** Anna-Lena Hoeger, Nico Jehmlich, Lydia Kipping, Carola Griehl, Matthias Noll

**Affiliations:** 1 Competence Center Algae Biotechnology, Anhalt University of Applied Sciences, Koethen, Germany; 2 Department of Molecular Systems Biology, Helmholtz-Centre for Environmental Research – UFZ GmbH, Permoserstr. 15, 04318 Leipzig, Germany; 3 Institute for Bioanalysis, Coburg University of Applied Sciences and Arts, Coburg, Germany; 4 Bayreuth Center of Ecology and Environmental Research (BayCEER), University of Bayreuth, Bayreuth, Germany

**Keywords:** Environmental microbiology, Industrial microbiology

## Abstract

The interactions of microalgae and their associated microbiomes have come to the fore of applied phycological research in recent years. However, the functional mechanisms of microalgal interactions remain largely unknown. Here, we examine functional protein patterns of the microalgae *Scenedesmus vacuolatus* and its associated bacterial community during algal infection by the endoparasite *Amoeboaphelidium protococcarum*. We performed metaproteomics analyses of non-infected (NI) and aphelid-infected (AI) *S.* *vacuolatus* cultures to investigate underlying functional and physiological changes under infectious conditions. We observed an increase in bacterial protein abundance as well as a severe shift of bacterial functional patterns throughout aphelid-infection in comparison to NI treatment. Most of the bacterial proteins (about 55%) upregulated in AI were linked to metabolism and transport of amino acids, lipids, coenzymes, nucleotides and carbohydrates and to energy production. Several proteins associated with pathogenic bacterial-plant interactions showed higher protein abundance levels in AI treatment. These functional shifts indicate that associated bacteria involved in commensalistic or mutualistic interactions in NI switch to opportunistic lifestyles and facilitate pathogenic or saprotrophic traits in AI treatment. In summary, the native bacterial microbiome adapted its metabolism to algal host die off and is able to metabolize nutrients from injured cells or decompose dead cellular material.

## Introduction

The industrial cultivation of microalgae has a wide range of academic opportunities and economic importance for various industrial sectors^1–3^. The worldwide production of fresh water microalgae is dominated by only a few species, mainly by the cyanobacterial genus *Arthrospira* and the green algal genus *Chlorella*, but also other microalgal species like *Scenedesmus* spp. gain increasing interest^4,5^. *Scenedesmus* species contain a diverse nutritional output and bioactive compounds with antioxidant and antimicrobial properties^6^. The versatility of *Scenedesmus* spp. is examined for human nutrition, aquaculture, bioremediation, cosmetics, pharmaceutical industries, and especially for bioenergy applications^7–9^.

However, like every other algae *Scenedesmus* cultures can be confronted by algal pathogens, which is a significant economic burden of microalgae production, but rarely reported^1,10–14^. The intrusion and integration of co-occurring organisms in industrial production plants are common, as sterile processing of algae cultures is economically and practically not feasible in mass production procedures^15,16^. Frequent biological contaminations include competitive algae and associated bacteria as well as predatory zooplankton, viruses, and parasitic fungi^1,14,17^. In industrial algae cultivation, algae-associated bacteria have long been regarded as mere contaminants. Bacterial contamination can indirectly compete for nutrients, or directly inactivate, attack or lyse algal cells^18–22^. However, in recent years many synergistic effects of interspecific interactions have been discovered and used in biotechnological applications^12,23^. The interactions of bacteria and algae are multidimensional and not only species-dependent but also affected by chemical and physical factors like temperature, nutrients, and light availability^24,25^. The relationship has to be therefore considered as a dynamic range of subsequent states of highly complex networks influenced by changing environmental conditions^21,23,24,26,27^.

Nevertheless, the metabolic level of algal interactions especially during parasitic invasion is still widely understudied. Quantitative proteomics of a B12-dependent algae grown in co-culture with bacteria on the one hand demonstrated the stability of the mutualism, but on the other hand indicated that *Lobomonas rostrata* experiences stress in co-culture with *Mesorhizobium loti*, and has to adjust its metabolism accordingly^28^. Proteomics analysis of the marine bacterium *Marinobacter adhaerens* and the diatom *Thalassiosira weissflogii* in co-culture suggest mutualistic interactions, depending on the release of amino acids by the diatom partner^29^. Furthermore, the findings of Krohn-Molt and colleagues^30^ imply that at least some of the triggers and signals involved in the microbial interaction with higher plants are already of relevance in algae-bacteria interactions. Plant-bacterial associations are highly diverse and involve various signaling molecules that influence host plants, which finally defines whether the outcome of the interaction is beneficial or harmful^31^.

The phylum Aphelidia is described as obligate endoparasites of various green algae, which pose a high threat for plant managers as they cause sudden and massive death of microalgal cells in natural environments as well as in industrial systems^11,13,17^. Many basic principles of aphelid infection are poorly understood, but the infection cycle of *Amoeboaphelidium protococcarum* has been described in detail^32^. After a host population is destroyed, aphelids aplanospores can remain in a dormant stadium until they come in contact with new host cells. In 2018, a global transcriptome of *Paraphelidium tribonemae* was employed to acknowledge the phagotrophic origin of fungi^33^. The results included a predicted proteome, covering full life-cycle data including cellulases, which were likely involved in algal cell-wall penetration, and enzymes involved in chitin biosynthesis, indicating typical fungal metabolism. The responses and defense reactions to pathogen infection of *A. protococcarum* on the green microalga *Graesiella emersonii* have just recently been investigated^34^. The defense of *G. emersonii* included pattern recognition receptors, large heat shock proteins, and reactive oxygen scavenging enzymes. In *A. protococcarum* genes for carbohydrate-active enzymes, pathogen-host interactions, putative effectors and vacuole transport including endocytosis, phagosome, ubiquitin-mediated proteolysis, and SNARE interactions, were significantly upregulated.

In previous work, we investigated the impact of infection with *A. protococcarum* on four algal hosts and discovered that during fungal invasion a profound shift in the composition of the associated bacterial microbiomes was observed^35^. The functional potential of compositional changes in the bacteria microbiome indicated an increase in hydrocarbon degradation and remediation when the algal host was attacked, but the detailed functional role of bacteria in the algae-based association remains unclear.

This work aims to further analyze the interactions of *S. vacuolatus* and their associated bacterial microbiome during aphelid infection by highlighting the underlying metabolic changes. Thereby, we focus on the functional patterns of co-occurring bacterial community members and compared aphelid-infected (AI) with non-infected (NI) *S. vacuolatus* cultures. We hypothesize that already present bacterial community members may shift their metaproteomic patterns. Since the release of algal-associated energetic compounds is expected during fungal infection, we assume that the availability of new compounds may trigger various bacterial degradation patterns. We questioned if the algal-associated bacterial community will adapt it’s metabolism and lifestyle to assure survival in the changing conditions caused by parasite infection.

## Results

### Algal growth parameters

**Figure 1:**
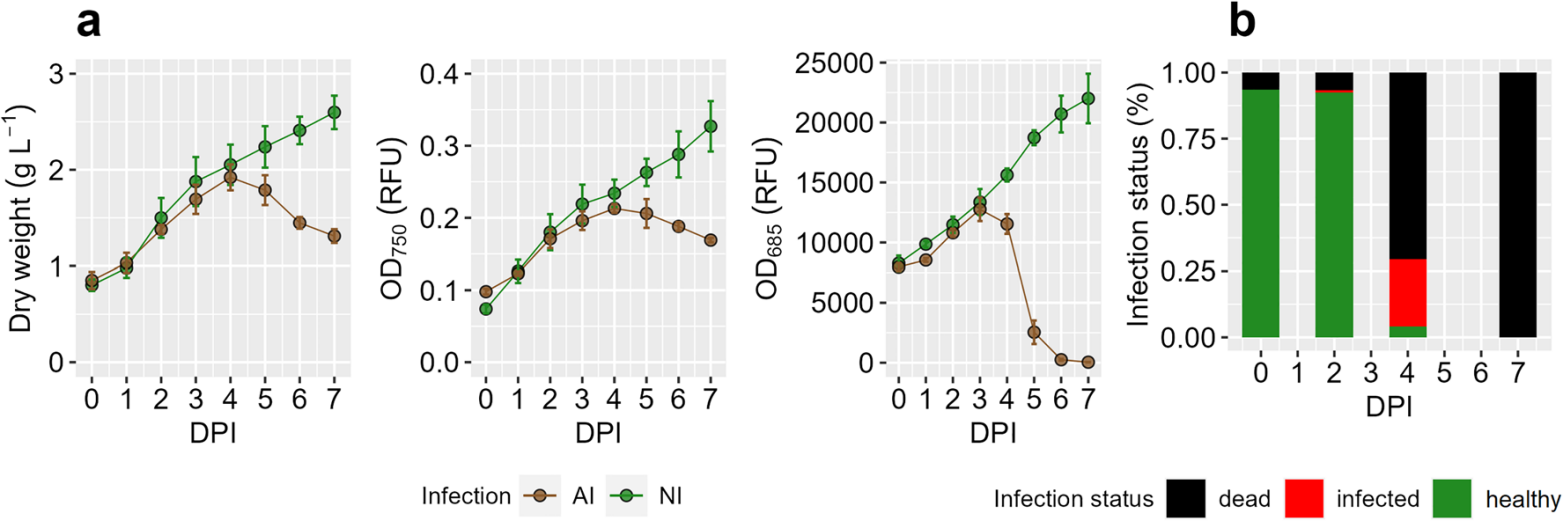
(**a**) Dry weight biomass content, optical density (OD_750_), normalized chlorophyll a fluorescence (OD_685_) of aphelid-infected (AI, brown) und non infected (NI, green) *S. vacuolatus* cultures over days post inoculation (DPI) (mean of n = 3 ± SD). (**b**) Mean of infection status was revealed by fluorescence microscopic observations from AI treatment over time (n = 3).

Chlorophyll *a* fluorescence (OD_685_) sharply decreased 3 days post inoculation (DPI), while dry weight and algal density (OD_750_) only slowly decreased 5 DPI in AI treatment compared to NI treatment (Fig. 1a). However, fluorescence microscopic analyses after wheat germ agglutinin (WGA) staining showed that algal infection was high even before 4 DPI and cell death of the algal population was completed 7 DPI (Fig. 1b).

### Metaproteomic composition

**Figure 2:**
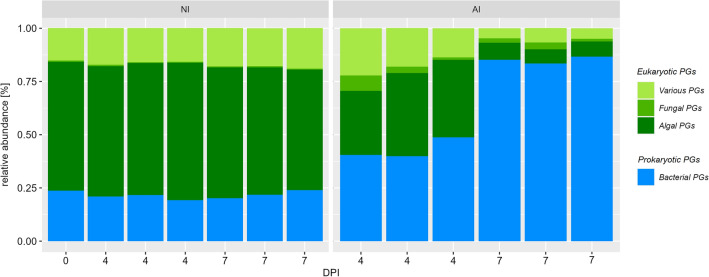
Composition of protein groups (PGs) according to taxonomic affiliation to bacterial (blue) and eukaryotic (algae = dark green, fungi = green, various = light green) PGs in non-infected (NI) and aphelid-infected (AI) *S. vacuolatus* cultures over days post inoculation (DPI). Mean of three independent replicates per treatment and incubation time are indicated (mean of n = 3 ± SD).

Overall, 1935 bacterial and 1786 eukaryotic Proteingroups (PGs) (1290 algal, 67 fungal and 468 various eukaryotic PGs) were observed. The taxonomic distribution of PGs differed significantly depending on presence of *A. protococcarum* and incubation time (Fig. 2). Proteomic patterns in NI treatment maintained similar over time and were clearly dominated by eukaryotic (~ 75%; mostly algal) and bacterial PGs (25%). In contrast, the overall number of algal and various PGs continuously decreased, while fungal PGs increased from 0.4% before infection to 3.6% mean relative abundance at 4 DPI and flattens to 2.1% at 7 DPI in AI treatment over time. Additionally, bacterial PGs steadily increased over time up to about 90% 7 DPI (Fig. 2).

**Figure 3:**
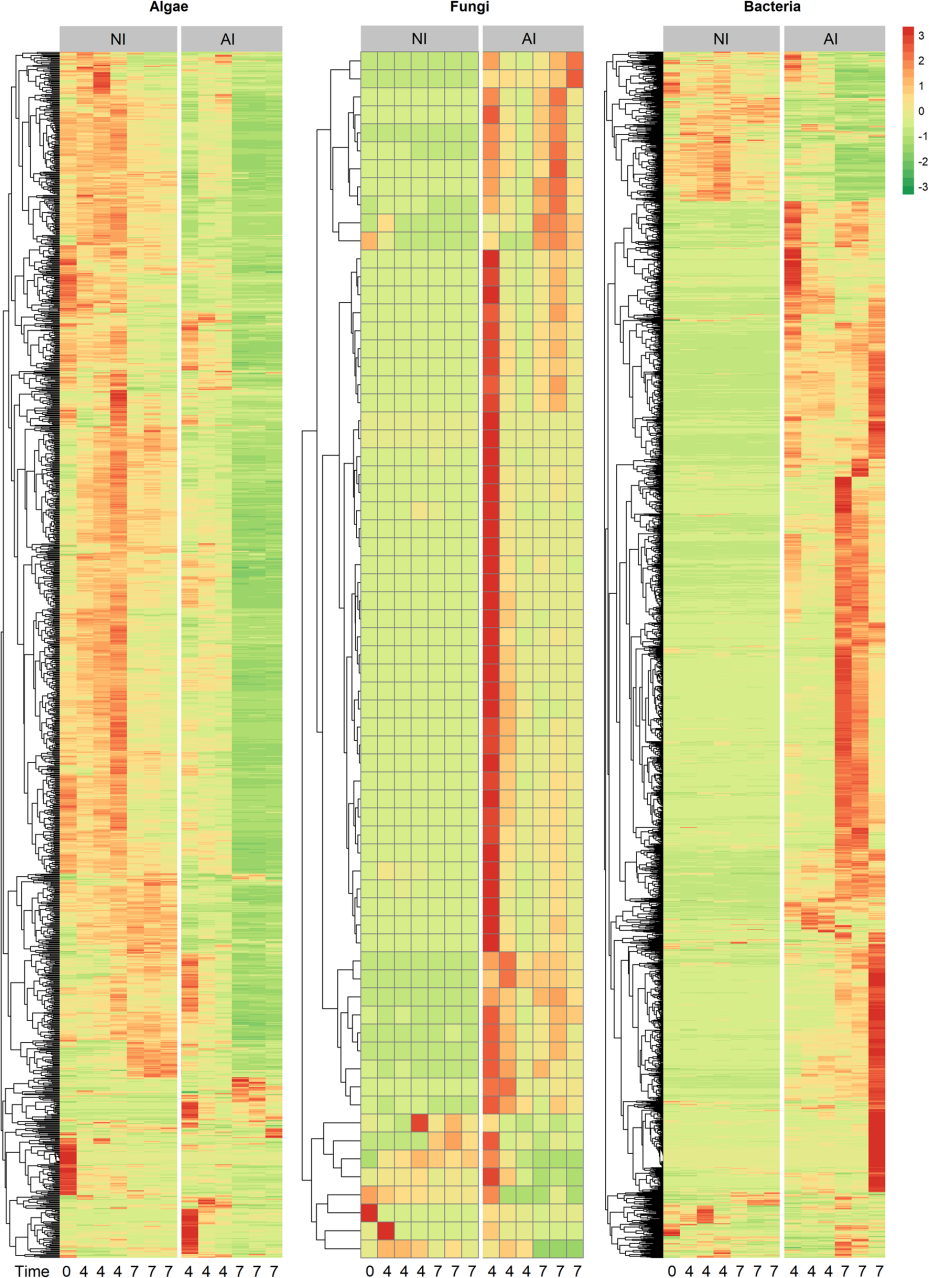
Mean of relative abundances of algal, fungal and bacterial protein groups (PGs) over time. Red colors indicate higher, while green colors indicate lower protein abundances of aphelid-infected (AI) and non-infected (NI) *S. vauolatus* cultures 0, 4 and 7 days post inoculation (DPI). The hetamp was calculuated in the open-source platform R (v3.6.1) with the pheatmap package v1.0.12 (RRID: SCR_016418)^55^.The tree based on PG presence represents the clustering of euclidean PG distances.

**Figure 4:**
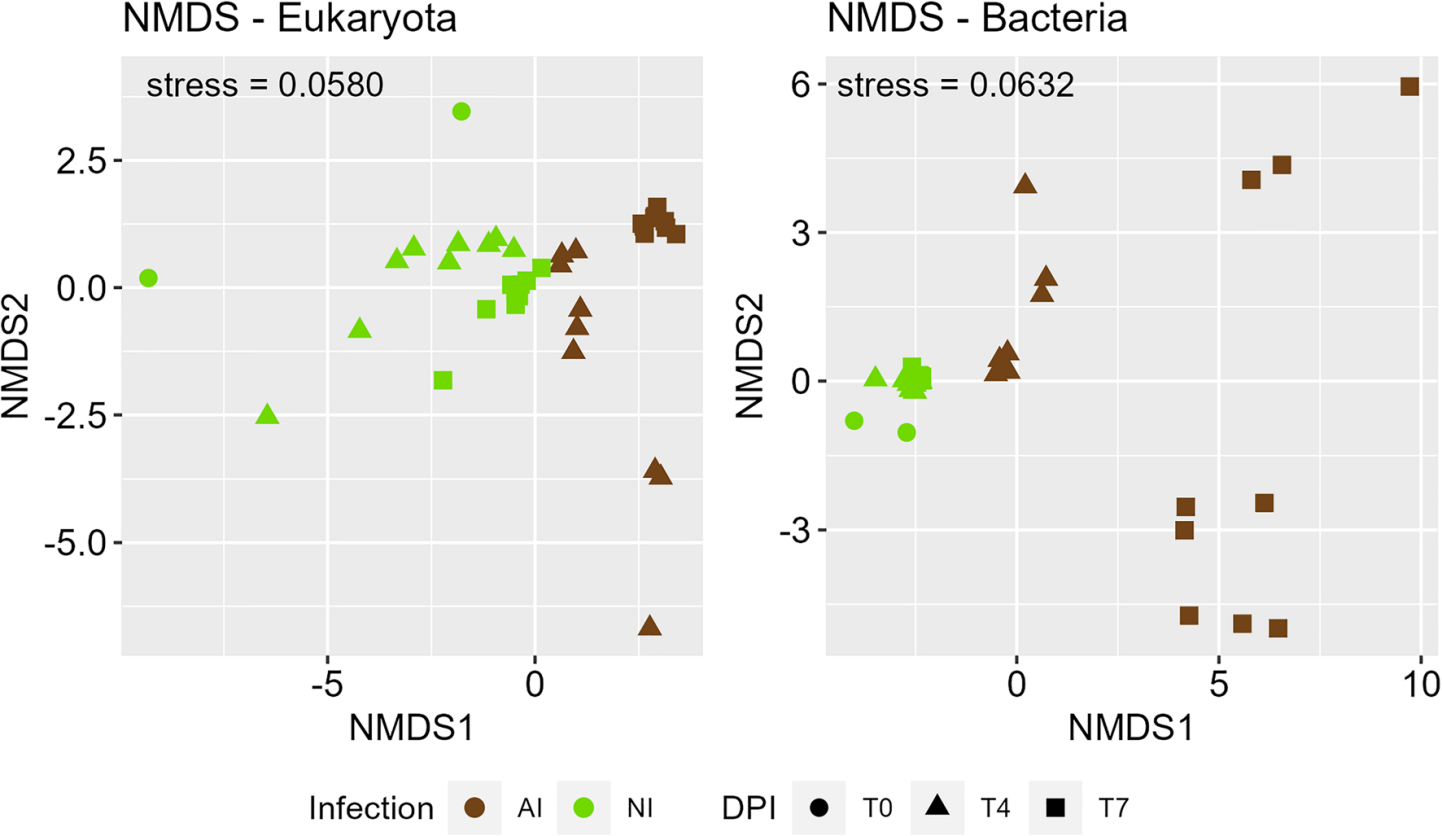
Nonmetric dimensional scaling (NMDS) based on euclidean distances of bacterial and eucaryotic protein group (PG) abundances. *S. vauolatus* cultures with aphelid infection (AI, brown) and without aphelid infection (NI, green) are denoted at the start of incubation (filled circle), after 4 (filled triangle) and 7 (filled square) days post inoculation (DPI)^54^. Ordination stresses are indicated.

The abundance of algal, fungal and bacterial PGs further underline distinct proteomic patterns between NI and AI treatment (Fig. 3). Algal PG abundances of NI treatment were similar between start 4 DPI and slightly changed 7 DPI. However, tremendous shifts in AI treatment were observed, and most algal PGs decreased in abundance. Though a small group of fungal PGs, that have very low abundance in NI treatment, exhibited high abundance in the AI treatment, especially in one replicate at 4 DPI. The course of infection was not exactly the same for all replicates at that timepoint causing this variation **(**Supplementary Fig. S1). In contrast, bacterial PGs showed complementary abundance patterns, if NI and AI treatment were compared (Fig. 3). Most bacterial PGs exhibited low abundances in NI and switched to high abundances in AI treatment, and vice versa. These shifts in eukaryotic and bacterial PG abundances were also found in the nonmetric dimensional scaling (NMDS), showing that PG patterns were highly treatment specific and changed severely in presence of *A. protococcarum* and incubation time (Fig. 4, Table 1).

**Table 1:** Effects of aphelid infection and incubation time on eukaryotic and bacterial protein groups as revealed by PERMANOVA analysis of euclidean distances.

Parameters	Eukaryota		Bacteria	
	R^2^	*p*-value	R^2^	*p*-value
Infection	0.36717	0.001*	0.29713	0.001*
Time	0.13853	0.001*	0.16647	0.001*
Residuals	0.49430		0.53641	

Significant differences (*p* < 0.05) are indicated by asterisk (*).

### Functional analysis

**Figure 5:**
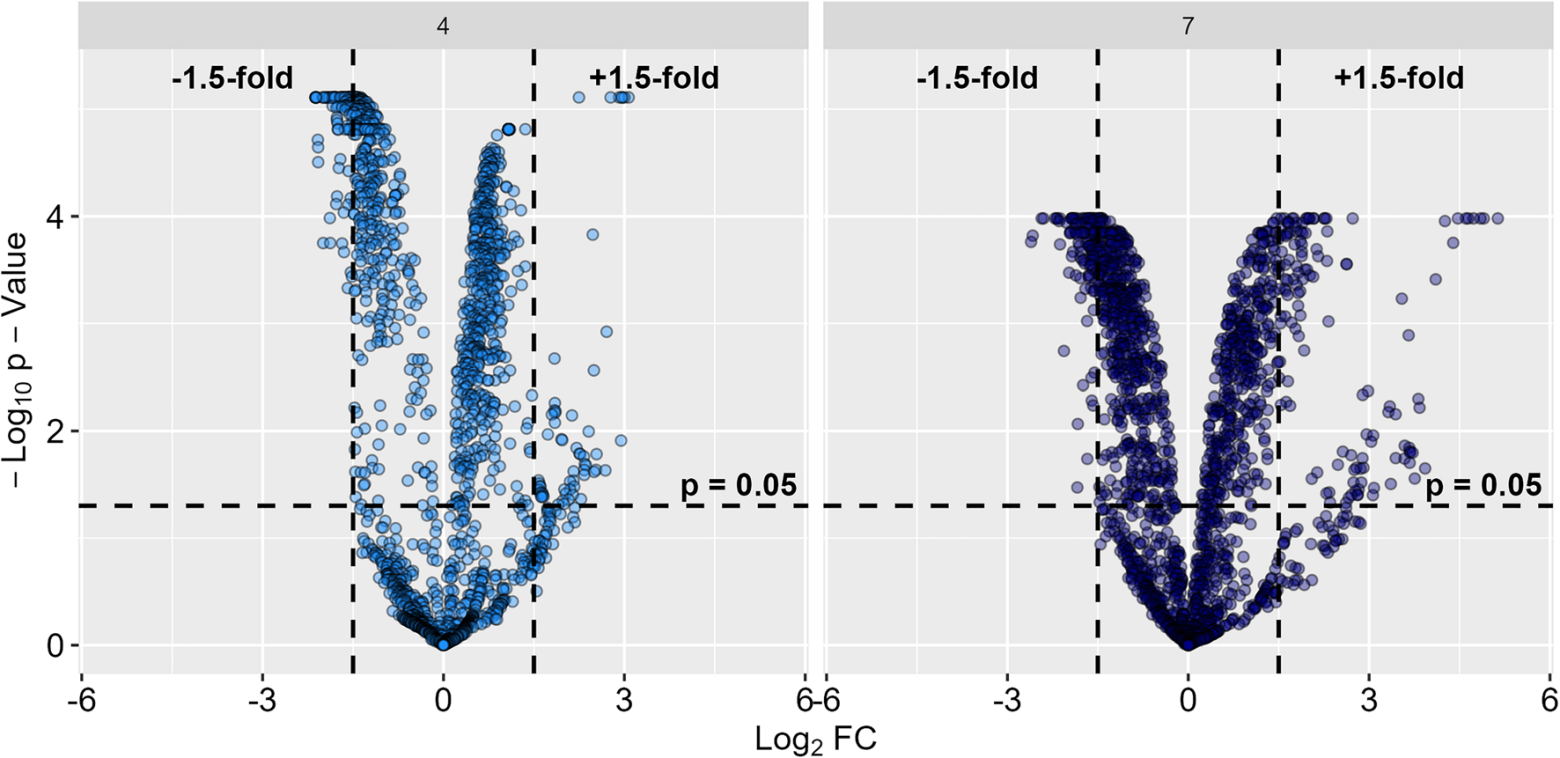
Volcano plot indicating differences in abundance of bacterial protein groups (PGs) between aphelid-infected (AI) and non-infected (NI) *S. vauolatus* cultures. PG fold changes (FCs) were calculated between AI and NI treatment after 4 days post inoculation (DPI) (light blue, left) and 7 DPI (dark blue, right)^56^. Log_2_ fold change (Log_2_FC) are plotted against − log_10_ transformed *p*-values to determine significantly (*p* > 0.05) upregulated (FC > 1.5) and downregulated (FC < − 1.5) PGs.

373 eukaryotic and 423 bacterial PGs were found with significant FC >  ± 1.5 between NI and AI treatment (Figs. 5 and S3). As bacterial PGs become predominant in AI treatment (Fig. 2) and their expression patterns tremendously changed (Fig. 3), we focused on the functional analyses of bacterial PGs during aphelid parasitosis while further details to eukaryotic PGs can be found in the supplementary information (Supplementary Figs. S3 and S4, Tables S1 and S2).

**Figure 6:**
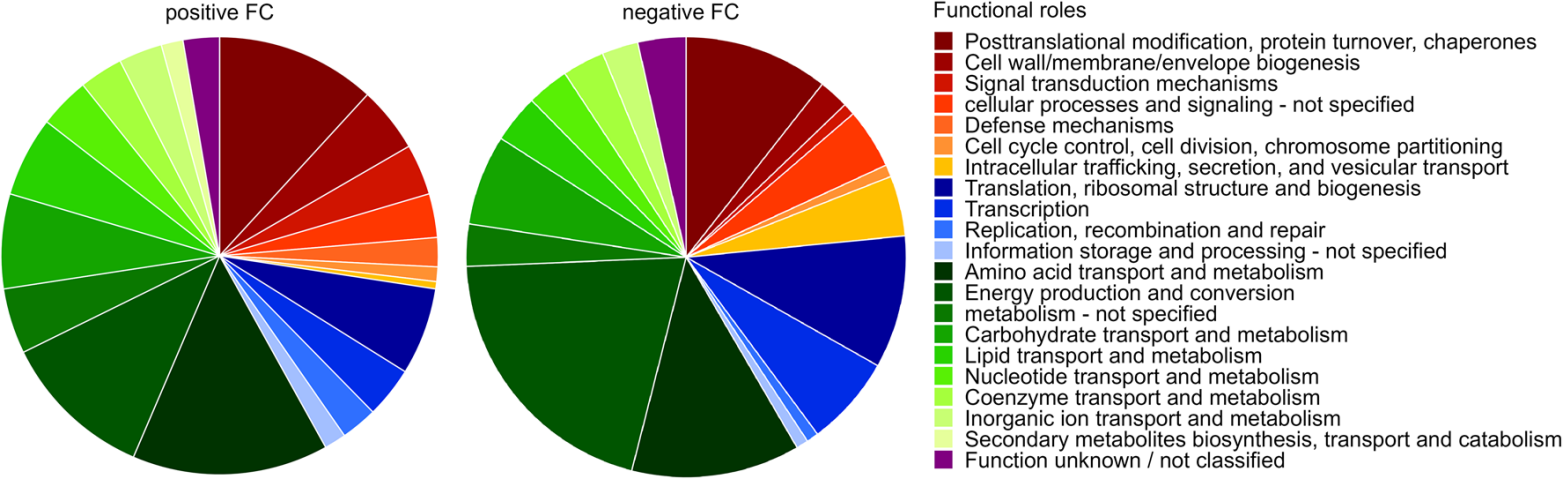
Functional shifts in bacterial protein groups (PGs) between aphelid-infected (AI) and non-infected (NI) *S. vauolatus* cultures with significantly upregulated (Fold changes (FC) > 1.5, left) and downregulated (FC < − 1.5, right) PGs. PGs were categorized into functional groups (green: metabolism; blue: transcription/translation; red: posttranslational modification, for details see figure legend) based on EggNOG database by Prophane. Relative proportions of each functional role were determined to be more (197 proteins) or less abundant (226 proteins) in AI treatment compared to NI treatment.

Main overexpressed bacterial protein groups (PGs) in AI treatment were predominantly assigned to ‘metabolism’ (55.4%), while ‘cellular processes and signalling’ accounted for 27.4% and ‘information storage and processing’ for 14.5% compared to NI treatment **(**Fig. 6). The functions of highly upregulated and abundant PGs were linked to metabolism and transport of amino acids, lipids, coenzymes, nucleotide and carbohydrate and energy production (Table 2). Other PGs concerning amino acids metabolism, energy production, posttranslational modifications as well as cell wall biogenesis were downregulated in AI treatment compared to NI treatment. Moreover, we found several upregulated PGs that were described to be involved in plant-bacterial interactions (Supplementary Table S2). For example, PGs from the transpeptidase-transglycosylase family, the outer membrane protein A precursor (ompA) and histidine kinases.

**Table 2:** List of most abundant (> 0.1%) bacterial protein groups (PGs) with fold changes (FC) > 1.5 with functional annotations, KEGG EC- and KO-numbers and Pfam accession numbers.

FC	Functional role	Functional subrole	Functional description	EC	KO	PFAM
1.55	Cellular processes and signaling	Posttranslational modification, protein turnover, chaperones	Heat shock 70 kDa protein	–	ko:K04043	PF00012
1.86	Information storage and processing	Transcription	Transcriptional regulator, LuxR family	–	ko:K13041	PF00196, PF00072
1.78	Information storage and processing	Replication, recombination and repair	Putative exonuclease SbcCD, C subunit	–	ko:K03546	PF13476, PF13558
1.75	Information storage and processing	Translation, ribosomal structure and biogenesis	This protein promotes the binding of aminoacyl-tRNA to the A-site of ribosomes during protein biosynthesis		ko:K02358	PF00009
1.66	Information storage and processing	Replication, recombination and repair	RNA helicase	3.6.4.13	ko:K12823	PF00270, PF00271
3.76	Metabolism	Metabolism - not specified	Reduction of activated sulfate into sulfite	1.8.4.10	ko:K00390	PF01507
2.95	Metabolism	Amino acid transport and metabolism	ABC-type branched-chain amino acid transport systems, periplasmic component	–	ko:K11959	PF13433
2.87	Metabolism	Coenzyme transport and metabolism	Tetrapolymerization of the monopyrrole PBG into the hydroxymethylbilane Porphobilinogen deaminase	2.5.1.61	ko:K01749	PF01379
2.73	Metabolism	Amino acid transport and metabolism	Argininosuccinate synthase	6.3.4.5	ko:K01940	PF00764
2.30	Metabolism	Carbohydrate transport and metabolism	Ribulose-1,5-bisphosphate carboxylase-oxygenase (RuBisCO) pathway	4.1.1.39	ko:K01601	PF00016
2.29	Metabolism	Nucleotide transport and metabolism	Carbamoyl-phosphate synthetase ammonia chain	6.3.5.5	ko:K01955	PF02786
1.95	Metabolism	Lipid transport and metabolism	Catalyzes the conversion of acetate into acetyl-CoA	6.2.1.1	ko:K01895	PF16177, PF00501
1.86	Metabolism	Energy production and conversion	Isocitrate/isopropylmalate dehydrogenases	1.1.1.42	ko:K00031	PF00180
1.66	Metabolism	Energy production and conversion	Glutamate/Leucine/Phenylalanine/Valine dehydrogenase	–	–	PF00208
1.53	Metabolism	Amino acid transport and metabolism	Cysteine synthase cystathionine beta—synthase family	2.5.1.47	ko:K01738	PF00291
1.79	Metabolism	Metabolism - not specified	Protease PfpI	–	–	PF01965

## Discussion

### Interactions of* S. vacuolatus* and* A. protococcarum*

Since fast destruction of algae cells due to aphelid infestation has been reported repeatedly^32,35,36^, a rapid decline of algae in the first days after infection was expected, which was also evident in our results (Fig. 1). Additionally, substantial PG abundance changes were found in AI treatment (Figs. 2, 3 and 4). Eukaryotic PGs continuously decreased (Fig. 2) during the destruction of the algal population (Fig. 1), while fungal PGs peaked with 4 DPI and flatten until 7 DPI. The abundance of fungal PGs matches the reported reproduction cycle of *A. protococcarum*, which includes intrusion of host cells, phagocytosis of the host cytoplasm and maturation of spores within 3–5 DPI^32,34^.

In contrast, both taxonomic distribution of PGs (Fig. 2) and the PG abundance patterns (Fig. 3) revealed high stability of algal and bacterial PGs, if algal partner was cultivated in NI treatment. Minor shifts in eukaryotic PG abundance patterns over time were observed (Fig. 3) which can be explained by algal cells entering senescent stages. Algal populations in batch cultures will undergo metabolic changes by emerging nutritional limitations^37–39^, resulting in metabolic adjustments of the algae.

### Change of the bacterial functional pattern in response to fungal infection of algal host culture

NI treatment was accompanied by low, but temporal stable bacterial PG patterns. The developing senescence of algae cells seems to have no substantial effect on bacterial PG abundances (Fig. 3). In contrast, we observed in AI treatment a severe increase of bacterial PG abundances over the course of aphelid infection in comparison to NI treatment (Fig. 2). Also, the bacterial functional patterns severely shifted (Fig. 3). In previous studies, we observed that *S. vacuolatus* had a very specific bacterial microbiome and that shifts in the composition of the associated microbiome were caused during aphelid infection^35^, which is in line with results of this study. Therefore, shifts in functional PG patterns (Fig. 3) were based on a change in both composition and metabolism of the indigenous microalgal-specific bacterial microbiome. We observed 423 bacterial PGs to be differently over- or underexpressed between NI and AI treatment (Fig. 5). Overall, PG abundances that were highly expressed in NI treatment such as cellular processes and signalling decreased, while metabolic PGs showed highest positive FCs in AI treatment. For example, pfpI protease was upregulated in AI treatment, facilitating the degradation of small peptides (Table 2). We also found ABC-type branched-chain amino acid transport system proteins to be upregulated, which are used for the uptake of a variety of small molecules including amino acids, metal ions, and sugars^40^. Furthermore, several enzymes catalysing the biosynthesis for amino acids like cysteine, leucine, arginine and pyrimidines were found. The conversion of acetate into acetyl-CoA (AcCoA) was upregulated to generate energy and biosynthetic components via the tricarboxylic acid cycle and the glyoxylate shunt, respectively (Table 2). The increase of these metabolic PGs (Fig. 6) indicates that the majority of associated bacterial community members adapted their metabolic patterns to utilize additional nutrients most likely from the algal biomass, which was released during aphelid infection. On the other hand, many PGs related to translation, transcription and posttranslational modifications were downregulated, indicating that adaptions in cellular processes and signalling were less important in AI treatment.

The increased abundance of several proteins related to bacterial pathogenic interactions with plant hosts were found (Supplementary Table S2). One example for proteins with increased PG abundance in AI treatment was the outer membrane protein A precursor (OmpA). The involvement of the *OmpA* gene in pathogenesis on different plants was reported for the bacterial species *Ralstonia solanacearum* and *Xanthomonas axonopodis*^41,42^. OmpA-mediated invasion was shown to be important in protein secretion during infection. Moreover, we found proteins from the histidine kinase family to be upregulated in AI treatment. Sensor histidine kinases were also described to be important in pathogenicity, as they were involved in a regulatory system as an essential factor for hypersensitive response and pathogenicity type III secretion system of *Burkholderia glumae* on rice plants^43^. Transpeptidase-transglycosylase was another high abundant bacterial PG in AI treatment. Multimodular transpeptidase-transglycosylase was identified as novel virulence factor of *Pseudomonas savastanoi* in olive knots^44^.

Conclusively, these results support the findings from Cirri and Pohnert^26^ that the relationship of microalgae and their associated microbiome is highly susceptible to changing external factors^26^. Healthy algal growth (NI) is characterized by stable algal–bacterial interaction patterns, but can be heavily affected by environmental disturbances like environmental stress or pathogen infections, which has been found also in other plant–microbe consortia ^45,46^. We observed that the bacterial community associated to *S. vacuolatus* was involved in commensal or mutualistic interactions with microalgae without aphelid infection (NI) and that it becomes opportunistic once aphelid infection (AI) occurred. The switch to a pathogenic lifestyle may be provoked by the destruction of the structural integrity of algal cells by aphelid intrusion. Algae exudates containing attractive nutrients or stress-induced effectors are set free, which caused an expression of pathogenic bacterial traits. On the other hand, injured or dead algal biomass can also be directly decomposed, which facilitated saprotrophic bacterial traits. These results highlight the critical importance of further studying the metabolome and understanding the basis of microbial interactions in algal populations to enhance the benefits of the natural microbiome in industrial microalgal cultivation. Future studies should focus on managing the associated bacterial community to enhance positive bacterial interactions prior algal infections. These studies ought to contain bacterial negative controls, which should be from researcher-assembled bacterial community composition previously isolated from the same algae host but without its host. In addition, absolute bacterial numbers should be taken into account to access not only qualitative but also quantitative shifts during algal infection.

## Material and methods

### Experimental design

Algal cultivation was performed in closed photobioreactors at the Competence Center Algal Biotechnology in Koethen, Germany in 2020. Algal strain *S. vacuolatus* SAG 211-8b was cultivated with autoclaved modified bolds basal medium^47^ (BBM) in bubble column reactors with 1.5 L capacity. The experiment was carried out at 23.5 °C, at a gas flow of 1.0 vvm (1% CO_2_) and permanent illumination at 100 μmol m^2^ s^−1^ with white LED light (380–750 nm). Bioreactors were inoculated to an optical density (OD_750_) of 0.2 (750 nm) under sterile conditions with 6 independent replicates, respectively. The preparation of aphelid inoculum from *A. protococcarum* strain AI15TR was set up as described earlier^35^. Shortly, cultures of *S. vacuolatus*, which contain a indigenous bacterial community^35^, were grown to mid-log phase in BBM for 5 days, diluted to a final OD_750_ of 0.2 in 1.4 L, infected with 100 mL *A. protococcarum* stock and cultivated in bubble column reactors using the same culture parameters applied in the following experiments. Seven days after infection, cultures were microscopically checked for infection status and frozen for later use. Three independent biological replicates of each algal culture were thereafter infected with 100 mL (6.6% vol./vol.) aphelid inoculum (AI), while 100 mL ddH_2_O was added to the additional three independent replicates as non-infected culture treatment (NI). Algal growth parameter like dry weight biomass (DW), optical density at 750 nm (OD_750_) and chlorophyll *a* fluorescence at 685 nm (OD_685_) were daily determined in independent triplicates. Determination of DW content was performed by filtering 5 mL culture suspension through glass microfiber filters (1.2 µm pore size)^48^. To determine OD_750_ algal suspension was measured photometrically at a wavelength of λ = 750 nm using an Infinite M200 Microplate Reader from Tecan (Tecan, Männedorf,Switzerland). On the same microplate reader OD_685_ was measured by excited the samples with a wavelength of 440 nm with a bandwidth of 9 nm and measured emissons at 685 nm with a bandwidth of 20 nm. OD_685_ was normalized by OD_750_ values to balance the influence of algal cell density on the results. Additionally, algal cells were stained with WGA and thereafter microscopically counted. The cell status was categorised as healthy (no signs of infection), infected (aphelid cysts and decreasing auto fluorescence) or dead (no auto fluorescence remained) (Supplementary Fig. S2). One-third (500 mL) of each algal culture was harvested by centrifugation (10.000×*g* for 5 min at room temperature) before, as well as on 4 and 7 DPI. Concentrated biomass for protein analysis was stored at − 80 °C until further processing.

### Protein extraction, quantification, and mass spectrometric analyses

Proteins were processed according to the workflow of Heyer and colleagues with few specific adaptions to algal biomass^49^. Shortly, proteins were extracted from 400 mg algae biomass by adding 1 g silica beads (0.5 mm), 700 µL liquid phenol (Carl Roth GmbH, Karlsruhe, Germany) and 400 μL 2 M sucrose solution and shaken in an MM200 ball mill (Retsch GmbH, Haan, Germany) for 20 min at 30 Hz. The upper phenolic phase was transferred and precipitated with the fourfold volume of ice-cold 0.1 M methanolic ammonium acetate for 60 min at − 20 °C. The pellet was washed twice with a threefold volume of ice-cold 80% acetone and 70% ethanol, respectively, and finally resuspended in 1 mL urea buffer (7 M urea, 2 M thiourea, 1% dithiothreitol). Protein concentrations were determined with Roti-Nanoquant dye (Carl Roth GmbH, Karlsruhe, Germany), which is based on a modified Bradford method, according to manufacturer’s protocol. The quantity and quality of the protein extracts were checked through a 12%/4%-gradient SDS-page. The tryptic digestion was realized by filter aided sample preparation (FASP) as described earlier^50^. Briefly, the protein extracts (50 µL in 200 µL urea buffer) were loaded onto 10 K Omega FASP filters (Pall Corporation, NY, USA), reduced and alkylated by addition of 100 µL dithiothreitol (20 min, 56 °C, 300×*g*) and 100 µL iodoacetamide (20 min, RT, 300 × g, in darkness). The FASP filter was washed for 2 min with 100 µL 8 M urea and three times with 100 µL 50 mM ammonium bicarbonate. 25 μg trypsin (Serva, Heidelberg, Germany) was dissolved in 25 μL 50 mM acetic acid and diluted to an enzyme to protein ratio of approximately 1:100 with 50 mM ammonium bicarbonate buffer (pH 7.9). Finally, FASP filters were incubated with 200 µL of trypsin solution (2 h, 37 °C, 300×*g*) and the peptide lysates were rinsed through the filter by the addition of 50 mM ammonium bicarbonate and 50 µL ultrapure water, respectively.

Peptide lysates were dissolved in 0.1% formic acid before liquid chromatography-mass spectrometry analysis (nanoLC-MS/MS). Peptide lysates (5 µL) were first loaded on the pre-column (µ-pre column, Acclaim PepMap, 75 µm inner diameter, 2 cm, C18, Thermo Scientific) for 5 min, at 4% mobile phase B (80% acetonitrile in nanopure water with 0.08% formic acid) and 96% mobile phase A (nanopure water with 0.1% formic acid), and then eluted from the analytical column (PepMap Acclaim C18 LC Column, 25 cm, 3 µm particle size, Thermo Scientific) over a 150 min linear gradient of mobile phase B (4–55% B).

Mass spectrometric analysis was performed on a Q Exactive HF mass spectrometer (Thermo Fisher Scientifc, Waltham, MA, USA) with a TriVersa NanoMate (Advion, Ltd., Harlow, UK) source in LC-chip coupling mode. Briefly, the mass spectrometer was set on loop count of 15 using for MS/MS scans with higher energy collision dissociation (HCD) at a normalized collision energy of 28%. MS scans were measured at a resolution of 120,000 in the scan range of 350–1600 m/z. MS ion count target was set to 3 × 10^6^ at an injection time of 120 ms. Ions for MS/MS scans were isolated in the quadrupole with an isolation window of 1.2 Da and were measured with a resolution of 30,000 in the scan range of 200–2000 m/z. The dynamic exclusion duration was set to 45 s with a 10 ppm tolerance. Automatic gain control target was set to 2 × 10^5^ with an injection time of 150 ms.

### Statistical data analysis

Proteome Discoverer (v2.5.0.400, Thermo Scientific) was used for protein identification and the MS/MS spectra acquired were searched with Sequest HT against the protein-coding bacterial sequences of the UniProt database^51^ (release 07/2021 for the bacterial taxa Oligoflexales, *Pseudomonas*, *Blastomonas*, *Brevundimonas*, *Devosia*, *Hydrogenophaga*, *Methylophilus*, *Sphingomonas*, *Stenotrophomonas*, *Variovorax*), the predicted proteome of *Paraphelidium tribonemae* released 11/2018^33^ and algae proteomes of the Alga-PrAS resource (release 06/2016). Selection of these data sets is based on the findings of our previous study^35^. Enzyme specificity was selected as trypsin with up to two missed cleavages allowed, using 10 ppm peptide ion tolerance and 0.02 Da MS/MS tolerances. Oxidation at methionines as the variable modifications and carbamidomethylation at cysteines as the static modification were selected. Only peptides with a false discovery rate (FDR) < 1% calculated by Percolator were considered as identified^52^. Identified proteins were grouped by applying the strict parsimony principle, in which protein hits were reported as the minimum set that accounts for all observable peptides. Protein abundances were calculated minora feature detector implemented in Proteome Discoverer. Taxonomical and functional annotation of observed PG were retrieved using the open-source software Prophane, searching NCBI for taxonomic and EggNOG (v5.0) database for functional annotations^53^. Protein annotations were calculated based on the lowest common ancestor approach of 0.6 per PG.

The retrieved prophane dataset was split up into bacteria and eukaryotes according to taxonomic assignment of PGs. PGs that could neither be affiliated to bacteria nor eukaryotes on superkingdom level were excluded from the analyses. To clearly assess the taxonomic composition during the course of infection, we classified eukaryotic PGs belonging to algal and fungal phyla. PGs that could not be assigned to a single taxon, were summarized into the last group which will hereafter be named ‘various’. NMDS on relative PG abundances based on Euclidian distances were computed with the vegan package on the open-source platform R (v3.6.1) on the eukaryotic and bacterial subsets^54^. Statistical significance of aphelid infection and incubation time were calculated using a PERMANOVA (adonis). PG abundances were log2 transformed and median standardized with the decostand function on both subsets respectively. Heatmaps on the log2-median transformed PG abundances were calculated using the pheatmap package v1.0.12 (RRID: SCR_016418)^55^. Differences in protein abundance levels between NI and AI treatment (Log_2_-Fold Changes = FC) were calculated using the R-package limma^56^. Data visualisation of FCs at 4 and 7 DPI as volcano plots was realized with ggplot2^57^. PGs with significant (*p* < 0.05) FC > 1.5 or < − 1.5 were extracted and proportions of functional subroles were plotted in pie charts of bacterial and eukaryotic PGs. Functional descriptions, EC numbers and Pfam accession numbers were checked for proteins that are typically associated with plant-pathogen interactions against the PHI database^58^.

## Supplementary Information

Supplementary Information.

## Data Availability

The datasets generated during and/or analyzed during the current study are available from the corresponding author on reasonable request.
