# Supplementary material for: Associated bacterial microbiome responds opportunistic once algal host Scenedesmus vacuolatus is attacked by endoparasite Amoeboaphelidium protococcarum
Source: Sci Rep. 2022 Aug 1;12:13187. doi: 10.1038/s41598-022-17114-1 (PMC9343445; doi:10.1038/s41598-022-17114-1)
Supplement: Supplementary file 1 — Supplementary Information. [file 41598_2022_17114_MOESM1_ESM.docx]

**Supplementary information to the manuscript: Associated bacterial microbiome reponds opportunistic once algal host *Scenedesmus vacuolatus* is attacked by endoparasite *Amoeboaphelidium protococcarum***

**Authors:** Anna-Lena Hoeger^1^, Nico Jehmlich^2^, Lydia Kipping^2^, Carola Griehl^1^, Matthias Noll^3,4*^

^1^Anhalt University of Applied Sciences, Competence Center Algae Biotechnology, Koethen, Germany

^2^Helmholtz-Centre for Environmental Research – UFZ GmbH, Department of Molecular Systems Biology, Permoserstr. 15, 04318 Leipzig, Germany

^3^Coburg University of Applied Sciences and Arts, Institute for Bioanalysis, Coburg, Germany

^4^Bayreuth Center of Ecology and Environmental Research (BayCEER), University of Bayreuth, Bayreuth, Germany

*Corresponding author: M. Noll ([matthias.noll@hs-coburg.de](mailto:matthias.noll@hs-coburg.de))


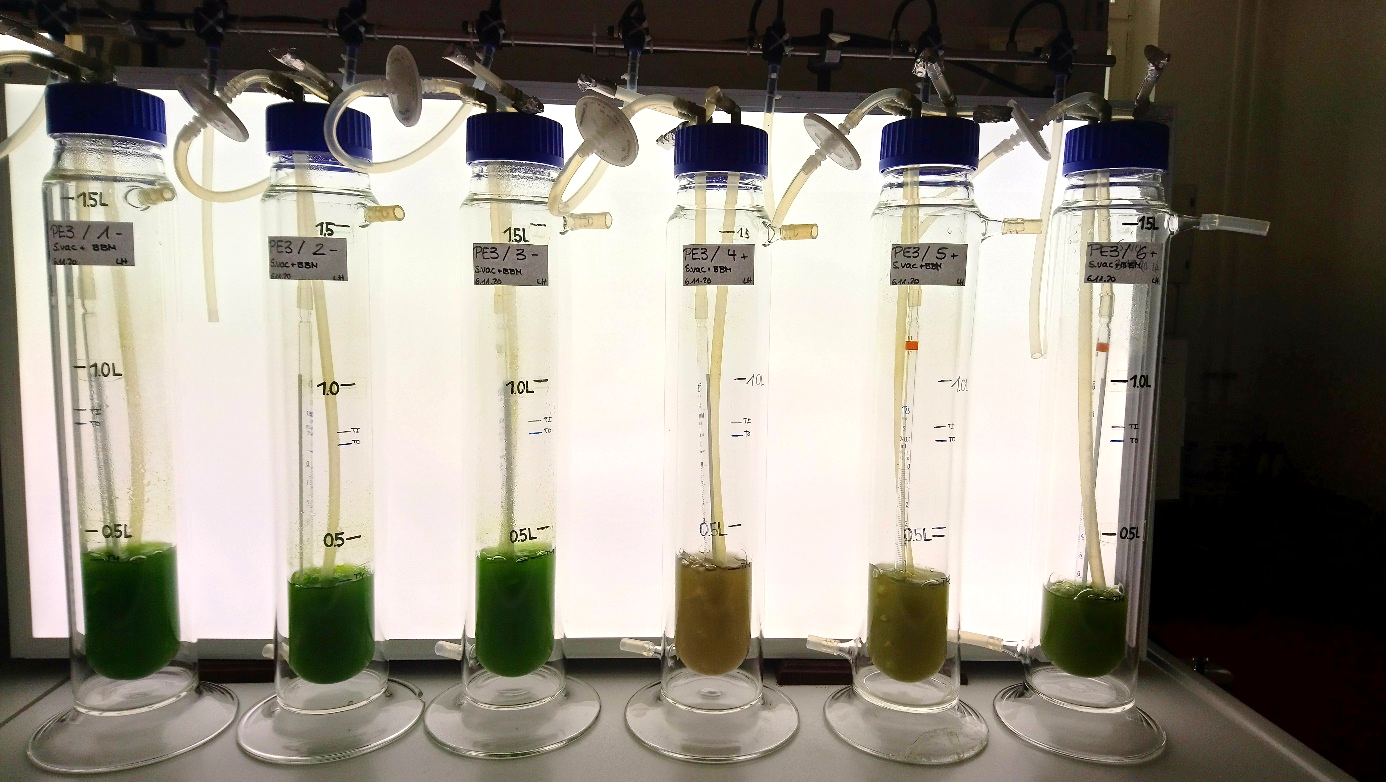


**Figure S1:** Experimental setup of *S. vacuolatus* cultures after 4 DPI with *A. protococcarum* . Each bubble column reactor is a replicate measurement, which were used for subsequent metaproteomic analyses.


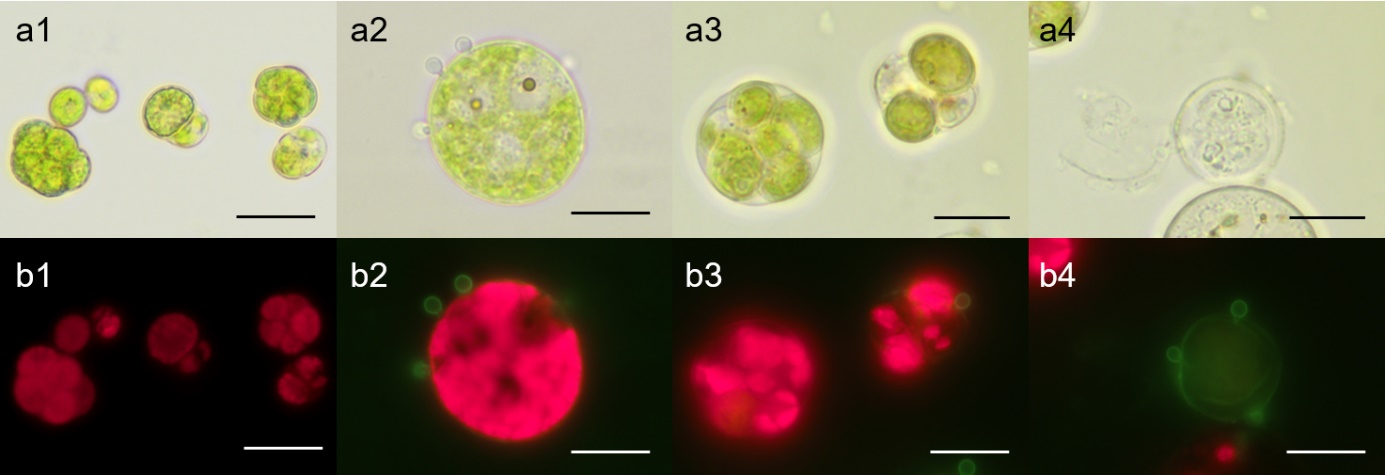


**Figure S2:** Microscopic images of the infection stages of *S. vacuolatus* cells with *A. protococcarum* of a) brightfield microscopy and b) fluorescent microcopy with WGA staining. 1) healthy non-infected *S. vacuolatus* cells with red autofluorescence, 2-3) infected *S. vacuolatus* cells with decreasing autofluorescence and green aphelid cycts and 4) dead *S. vacuolatus* cell with mounted aphelid cysts and almost no auto fluorescence.


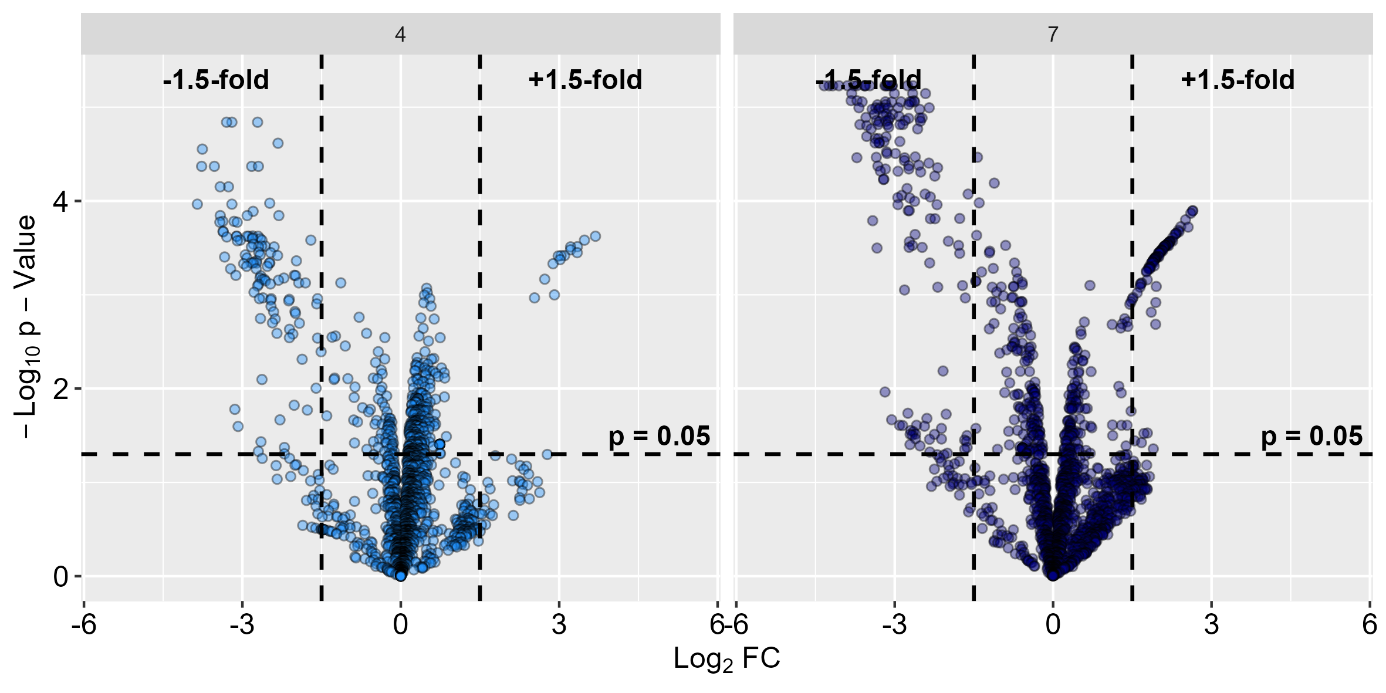


**Figure S3:** Volcano plot of euakryotic protein groups (PGs) between aphelid infected (AI) and non-infected (NI) *S. vauolatus* cultures. PG fold changes (FCs) were calculated between AI and NI treatment after four day of incubation (light blue, left) and seven days of incubation (dark blue, right). Log_2_ fold change (Log_2_FC) are plotted against -log_10_transformed p-values to determine significantly (p>0.05) upregulated (FC>1.5) and downregulated (FC < -1.5).


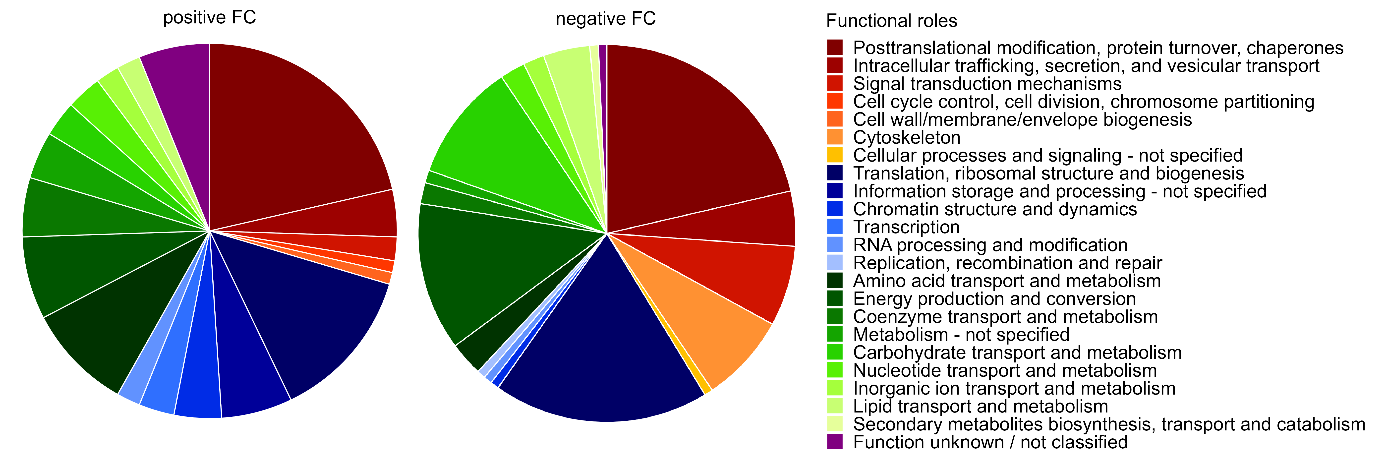


**Figure S4:** Functional shift in eukaryotic PGs between aphelid infected (AI) and non-infected (NI) treatment with fold changes > +1.5 (left pie chart) and >-1.5 (right pie chart). PGs were categorized into functional groups (green: metabolism; blue: transcription/translation; brown: posttranslational modification, for details see figure legend) based on EggNOG datadabse by prophane. Relative proportions of each functional category determined to be more (98 proteins) or less abundant (276 proteins) in AI treatment compared to NI treatment.

**Table S1:** List of 15 most abundant eukaryotic Protein groups (PGs) with fold changes > ±1.5 with functional annotations and KEGG EC- and KO-numbers and Pfam accessions

| **logFC** | **Functional role** | **Functional subrole** | **Functional description** | **EC-Nr.** | **KO-Nr.** | **PFAM** |
| --- | --- | --- | --- | --- | --- | --- |
| -4.34 | Metabolism | Nucleotide transport and metabolism | Nucleoside diphosphate kinase | 2.7.4.6 | K00940 | NDK |
| -4.24 | Cellular processes and signaling | Cytoskeleton | ATP binding | - | K05692 | Actin |
| -4.17 | Cellular processes and signaling | Posttranslational modification, protein turnover, chaperones | Belongs to the peptidase S8 family | 3.4.21.48 | K01336 | Peptidase_S8 |
| -4.06 | Metabolism | Carbohydrate transport and metabolism | Glyceraldehyd-3-phosphate dehydrogenase | 1.2.1.12 | K00134 | Gp_dh_C |
| -3.85 | Cellular processes and signaling | Cytoskeleton | ATP binding | - | K05692 | Actin |
| -3.77 | Metabolism | Nucleotide transport and metabolism | Nucleoside diphosphate kinase | 2.7.4.6 | K00940 | NDK |
| -3.76 | Cellular processes and signaling | Posttranslational modification, protein turnover, chaperones | Belongs to the peptidase S8 family | 3.4.21.48 | K01336 | Peptidase_S8 |
| -3.53 | Metabolism | Carbohydrate transport and metabolism | Glyceraldehyd-3-phosphate dehydrogenase | 1.2.1.12 | K00134 | Gp_dh_C |
| -3.14 | Cellular processes and signaling | Posttranslational modification, protein turnover, chaperones | C-terminal domain of 1-Cys peroxiredoxin | 1.11.1.15 | K03386 | 1-cysPrx_C |
| -3.08 | Metabolism | Nucleotide transport and metabolism | Nucleoside diphosphate kinase | 2.7.4.6 | K00940 | NDK |
| -2.71 | Information storage and processing | Translation, ribosomal structure and biogenesis | Promotes the GTP-dependent binding of aminoacyl-tRNA to the A-site of ribosomes during protein biosynthesis | - | K03231 | GTP_EFTU |
| -2.65 | Cellular processes and signaling | Posttranslational modification, protein turnover, chaperones | C-terminal domain of 1-Cys peroxiredoxin | 1.11.1.15 | K03386 | 1-cysPrx_C |
| -2.52 | Metabolism | Nucleotide transport and metabolism | Nucleoside diphosphate kinase | 2.7.4.6 | K00940 | NDK |
| -1.66 | Metabolism | Carbohydrate transport and metabolism | Fructose-bisphosphate aldolase | 4.1.2.13 | K01623 | Glycolytic |
| -1.58 | Information storage and processing | Translation, ribosomal structure and biogenesis | Promotes the GTP-dependent binding of aminoacyl-tRNA to the A-site of ribosomes during protein biosynthesis | - | K03231 | GTP_EFTU |

**Table S2:** List of bacterial Protein groups (PGs) with fold changes > 1.5 playing a role in plant- bacterial interactions according to the plant-pathogen database (PHI)

| **PG** | **DPI** | **Description** | **EC** | **KEGG_ko** | **PFAM** | **Mutant Phenotyp** | **Pathogen** | **Host** | **ProteinID** | **PHI_ID** |
| --- | --- | --- | --- | --- | --- | --- | --- | --- | --- | --- |
| 395 | 7 | Part of the ABC transporter complex ModABC involved in molybdenum import. Responsible for energy coupling to the transport system | 3.6.3.29 | ko:K02017 | PF00005 | reduced virulence | *Pseudomonas cannabina* | *Avena strigosa* | F3HSJ9 | PHI:9661 |
| 781 | 7 | Phospho-2-dehydro-3-deoxyheptonate aldolase | 2.5.1.54 | ko:K01626 | PF01474 | loss of pathogenicity | *Ralstonia solanacearum* | *Solanum lycopersicum* | Q8XW15 | PHI:8884 |
| 1656 | 7 | Carbamoyl-phosphate synthetase ammonia chain | 6.3.5.5 | ko:K01955 | PF02786 | reduced virulence | *Pseudomonas syringae* | *Solanum lycopersicum* | Q87WP3 | PHI:6594 |
| 1809 | 7 | Acts as a processive, ATP-dependent zinc metallopeptidase for both cytoplasmic and membrane proteins. Plays a role in the quality control of integral membrane proteins | - | ko:K03798 | PF00004 | reduced virulence | *Erwinia amylovora* | *Malus domestica* |  | PHI:11094 |
| 1815 | 4 | Belongs to the thiolase family | 2.3.1.16,  2.3.1.9 | ko:K00626,  ko:K00632 | PF02803 | reduced virulence | *Leptosphaeria maculans* | *Brassica napus* | Q0QWD8 | PHI:598 |
| 1931 | 4 | Catalyzes the final step of fatty acid oxidation in which acetyl-CoA is released and the CoA ester of a fatty acid two carbons shorter is formed | 2.3.1.9 | ko:K00626 | PF02803 | reduced virulence | *Leptosphaeria maculans* | *Brassica napus* | Q0QWD8 | PHI:598 |
| 1931 | 4 | Belongs to the thiolase family | 2.3.1.9 | ko:K00626 | PF02803 | reduced virulence | *Leptosphaeria maculans* | *Brassica napus* | Q0QWD8 | PHI:598 |
| 2385 | 7 | Belongs to the amidase family | 3.5.1.4,  6.3.5.6,  6.3.5.7 | ko:K01426,  ko:K02433 | PF01510 | reduced virulence | *Pseudomonas cannabina* | *Avena strigosa* | F3HQR8 |  |
| 2407 | 7 | HsdM N-terminal domain | 2.1.1.72 | ko:K03427 | PF12161 | reduced virulence | *Xanthomonas axonopodis* | *Glycine max* | A0A1D9ELP8 | PHI:8921 |
| 2781 | 7 | Glycosyl transferases group 1 | - | - | PF00534 | unaffected pathogenicity | *Pseudomonas syringae* | *Arabidopsis thaliana* | Q888F9 | PHI:9688 |
| 2781 | 4 | Glycosyl transferases group 1 | - | - | PF00534 | unaffected pathogenicity | *Pseudomonas syringae* | *Arabidopsis thaliana* | Q888F9 | PHI:9688 |
| 2802 | 7 | Two component transcriptional regulator, LuxR family | - | ko:K13041 | PF00196 | loss of pathogenicity | *Pseudomonas syringae* | *Phaseolus vulgaris* | Q93NJ9 | PHI:6105 |
| 2837 | 7 | Putative diguanylate phosphodiesterase | - | - | PF00563 | increased virulence | *Burkholderia cenocepacia* | *Galleria mellonella* | B4E974 | PHI:8841 |
| 2877 | 7 | Histidine kinase | 2.7.13.3 | ko:K02484,  ko:K07645 | PF08521 | reduced virulence | *Xanthomonas campestris* | *Brassica oleracea* | B0RY65 | PHI:3107 |
| 2915 | 7 | Peptidoglycan polymerase that catalyzes glycan chain elongation from lipid-linked precursors | 2.4.1.129 | ko:K03814 | PF00912 | reduced virulence | *Pseudomonas savastanoi* | *Olea europaea* | D7HWI3 | PHI:2776 |
| 3524 | 4 | Catalyzes the synthesis of acetoacetyl coenzyme A from two molecules of acetyl coenzyme A. It can also act as a thiolase, catalyzing the reverse reaction and generating two-carbon units from the four-carbon product of fatty acid oxidation | 2.3.1.9 | ko:K00626 | PF02803 | reduced virulence | *Leptosphaeria maculans* | *Brassica napus* | Q0QWD8 | PHI:598 |
| 3561 | 4 | ABC transporter | 3.6.3.17 | ko:K10545 | PF00005 | reduced virulence | *Pseudomonas cannabina* | *Avena strigosa* | F3HSJ9 | PHI:9661 |
| 3625 | 7 | Type VI secretion protein, vc_a0107 family | - | ko:K11901 | PF05591 | reduced virulence | *Ralstonia solanacearum* | *Solanum melongena* |  | PHI:11069 |
| 3654 | 7 | Histidine kinase | - | - | PF08521 | reduced virulence | *Xanthomonas campestris* | *Brassica oleracea* | B0RY65 | PHI:3107 |
| 3691 | 7 | Transglycosylase | - | - | PF00912 | reduced virulence | *Pseudomonas savastanoi* | *Olea europaea* | D7HWI3 | PHI:2776 |
| 3703 | 7 | Belongs to the NAD(P)-dependent epimerase dehydratase family | 5.1.3.2 | ko:K01784 | PF01370 | reduced virulence | *Pectobacterium carotovorum* | *Daucus carota* |  | PHI:9304 |
| 3722 | 4 | Belongs to the resistance-nodulation-cell division (RND) (TC 2.A.6) family | - | - | PF00873 | reduced virulence | *Pseudomonas cannabina* | *Brassica oleracea* | F3HJT6 | PHI:9660 |
| 3731 | 4 | Beta-lactamase superfamily domain | - | - | PF12706 | reduced virulence | *Xanthomonas campestris* | *Raphanus sativus* | Q4UWM4 | PHI:3965 |
| 3749 | 7 | PFAM OmpA MotB domain protein | - | - | PF01389 | reduced virulence | *Xanthomonas axonopodis* | *Glycine max* |  | PHI:9221 |

**Table S3:** List of eukaryotic Protein groups (PGs) with fold changes > 1.5 playing a role in plant- fungal interactions according to the plant-pathogen database (PHI)

| **PG** | **DPI** | **Description** | **EC** | **KEGG_ko** | **PFAM** | **Mutant Phenotype** | **Pathogen** | **Host** | **ProteinID** | **PHI_ID** |
| --- | --- | --- | --- | --- | --- | --- | --- | --- | --- | --- |
| 733 | 7 | Leucine Rich repeat | - | - | [PF13516](http://pfam.xfam.org/family/PF13516) | effector | *Phytophthora capsici* | *Nicotiana benthamiana* |  | PHI:7029 |
| 789 | 7 | The proteasome is a multicatalytic proteinase complex which is characterized by its ability to cleave peptides with Arg, Phe, Tyr, Leu, and Glu adjacent to the leaving group | 3.4.25.1 | ko:K02734 | [PF00227](http://pfam.xfam.org/family/PF00227) | effector | *Hyaloperonospora arabidopsidis* | *Arabidopsis thaliana* | G3C9P1 | PHI:4751 |
| 1419 | 7 | Belongs to the glyceraldehyde-3-phosphate dehydrogenase family | 1.2.1.13 | ko:K05298 | PF02800 | reduced virulence |  |  |  |  |
| 1450 | 7 | Aldehyde dehydrogenase family | 1.2.1.3 | ko:K00128 | PF00171 | reduced virulence |  |  |  |  |
| 3327 | 4 | belongs to the protein kinase superfamily (Autophagy) | 2.7.11.17 | ko:K07359 | [PF00069](http://pfam.xfam.org/family/PF00069) | reduced virulence | *Ustilago maydis* | *Zea mays* | O59918 | PHI:158 |
| 3150 | 4 | Zn-dependent metallohydrolase RNA specificity domain | - | - | PF00753, PF07521 | reduced virulence | *Xanthomonas campestris* |  |  |  |
| 489 | 7 | lactate/malate dehydrogenase, NAD binding domain | 1.1.1.37 | ko:K00026 | PF02866 | reduced virulence | *Fusarium graminearum* | *Triticum aestivum* | I1R9K2 | PHI:8645 |
| 727 | 7 | Sulfite reductase | 1.8.7.1 | ko:K00392 | PF01077 | unaffected pathogenicity | *Erwinia amylovora* | *Malus domestica* | D4HWM2 | HI:9323 |
| 792 | 7 | PUB domain | - | - | PF09409, PF00627 | reduced virulence | *Magnaporthe oryzae* | *Oryza sativa* | G4NGN1 | PHI:11061 |
| 796 | 7 | ubiquitin-ubiquitin ligase activity | 2.1.3.15, 2.3.2.27,  6.4.1.2 | ko:K01962, ko:K10597,  ko:K12169 | PF00622, PF04564, PF10408 | reduced virulence | *Magnaporthe oryzae* | *Oryza sativa* | G4MVC5 | PHI:6927 |
| 877 | 7 | Belongs to the ubiquitin-activating E1 family | 6.2.1.45 | ko:K03178 | PF16191, PF00899, PF10585 | reduced virulence | *Magnaporthe oryzae* | *Oryza sativa* | G4MVC5 | PHI:6927 |
| 968 | 7 | ABC transporter | 3.6.3.25 | ko:K06020 | [PF00005](http://pfam.xfam.org/family/PF00005) | reduced virulence | *Fusarium graminearum* | *Triticum aestivum* | I1S2J9 | PHI:3924 |
| 971 | 7 | protein disulfide oxidoreductase activity | 1.8.4.9 | ko:K05907 | PF01507, PF01323 | reduced virulence | *Botrytis cinerea* | *Phaseolus vulgaris* |  | PHI:3079 |
| 1079 | 7 | Belongs to the TRAFAC class dynamin-like GTPase superfamily. Dynamin Fzo YdjA family | - | - | PF01031, PF02212 | reduced virulence | *Fusarium graminearum* | *Triticum aestivum* | I1RBC0 | PHI:10481 |
| 1136 | 4 | Serine threonine-protein phosphatase | 3.1.3.16 | ko:K15498 | PF00149 | reduced virulence | *Fusarium oxysporum* | *Solanum lycopersicum* | A0A0D2XC66 | PHI:10879 |
| 1136 | 7 | Serine threonine-protein phosphatase | 3.1.3.16 | ko:K15498 | [PF00149](http://pfam.xfam.org/family/PF00149) | reduced virulence | *Fusarium oxysporum* | *Solanum lycopersicum* | A0A0D2XC66 | PHI:10879 |
| 1348 | 7 | SCP-2 sterol transfer family | - | - | PF02036 | reduced virulence | *Ustilago maydis* | *Zea mays* | A0A0D1E3D1 | PHI:8183 |
| 1408 | 7 | Ubiquitinyl hydrolase 1 | 3.4.19.12 | ko:K11836 | PF00627, PF00443 | reduced virulence | *Magnaporthe oryzae* | *Oryza sativa* | G4NGN1 | PHI:11061 |
| 1441 | 7 | Thioredoxin-like domain | 5.3.4.1 | ko:K09580 | PF00085 | reduced virulence | *Botrytis cinerea* | *Phaseolus vulgaris* |  | PHI:3081 |
| 3275 | 7 | Belongs to the sirtuin family. Class I subfamily | - | ko:K11412 | [PF02146](http://pfam.xfam.org/family/PF02146) | reduced virulence | *Magnaporthe oryzae* | *Oryza sativa* | G4NHH2 | PHI:11101 |
